# Decoding of Covert Vowel Articulation Using Electroencephalography Cortical Currents

**DOI:** 10.3389/fnins.2016.00175

**Published:** 2016-05-03

**Authors:** Natsue Yoshimura, Atsushi Nishimoto, Abdelkader Nasreddine Belkacem, Duk Shin, Hiroyuki Kambara, Takashi Hanakawa, Yasuharu Koike

**Affiliations:** ^1^Precision and Intelligence Laboratory, Tokyo Institute of TechnologyYokohama, Japan; ^2^Department of Functional Brain Research, National Center of Neurology and Psychiatry, National Institute of NeuroscienceTokyo, Japan; ^3^Department of Advanced Neuroimaging, Integrative Brain Imaging Center, National Center of Neurology and PsychiatryTokyo, Japan; ^4^Department of Neurosurgery, Osaka University Medical SchoolOsaka, Japan; ^5^Department of Electronics and Mechatronics, Tokyo Polytechnic UniversityAtsugi, Japan; ^6^Precursory Research for Embryonic Science and Technology, Japan Science and Technology AgencyTokyo, Japan; ^7^Solution Science Research Laboratory, Tokyo Institute of TechnologyYokohama, Japan

**Keywords:** brain-computer interfaces, silent speech, electoencephalography, functional magnetic resonance imaging, inverse problem

## Abstract

With the goal of providing assistive technology for the communication impaired, we proposed electroencephalography (EEG) cortical currents as a new approach for EEG-based brain-computer interface spellers. EEG cortical currents were estimated with a variational Bayesian method that uses functional magnetic resonance imaging (fMRI) data as a hierarchical prior. EEG and fMRI data were recorded from ten healthy participants during covert articulation of Japanese vowels /a/ and /i/, as well as during a no-imagery control task. Applying a sparse logistic regression (SLR) method to classify the three tasks, mean classification accuracy using EEG cortical currents was significantly higher than that using EEG sensor signals and was also comparable to accuracies in previous studies using electrocorticography. SLR weight analysis revealed vertices of EEG cortical currents that were highly contributive to classification for each participant, and the vertices showed discriminative time series signals according to the three tasks. Furthermore, functional connectivity analysis focusing on the highly contributive vertices revealed positive and negative correlations among areas related to speech processing. As the same findings were not observed using EEG sensor signals, our results demonstrate the potential utility of EEG cortical currents not only for engineering purposes such as brain-computer interfaces but also for neuroscientific purposes such as the identification of neural signaling related to language processing.

## Introduction

Brain-computer interface (BCI) spellers offer a means of hands-free character input for individuals with motor impairments through the utilization of brain activity signals (Kubler et al., 2001; Wolpaw et al., 2002; Birbaumer, 2006a,b; Birbaumer and Cohen, 2007; Shih et al., 2012). Most BCI spellers use distinct electroencephalography (EEG) activity such as the P300 event-related potential (Farwell and Donchin, 1988; Nijboer et al., 2008) or steady-state visual evoked potentials (SSVEP) (Cheng et al., 2002). The P300 is a positive peak potential which appears approximately 300 ms after stimulus onset in reaction to infrequently presented visual or auditory stimuli (Sutton et al., 1967), whereas SSVEPs are generated in reaction to high-speed flashing light and are characterized by sinusoidal-like waveforms with frequencies synchronized to those of the flashing light (Adrian and Matthews, 1934).

BCI spellers based on the P300 and SSVEPs are considered “reactive” BCI spellers, since they utilize potentials arising in reaction to external stimuli, such as the appearance of a desired character on a communication board. Conversely, “active” BCI spellers are spellers that utilize brain activity consciously controlled by the user (Zander et al., 2010), like that when imagining a vowel. As such, active BCI spellers are not subject to limitations associated with providing external stimuli (e.g., time and space required to display a character). Comparing these spellers, reactive BCI spellers are closer to the market because of their higher information transfer rates and stability. However, with developments in neuroimaging, active BCI spellers have drawn attention from researchers using neural decoding techniques. EEG has been used to decode English vowels /a/ and /u/ (Dasalla et al., 2009); Dutch vowels /a/, /i/, and /u/ (Hausfeld et al., 2012); words “yes” and “no” (Lopez-Gordo et al., 2012); and Chinese characters for “left” and “one” (Wang et al., 2013). Decoding performance in these studies were higher than chance level but not comparable to reactive BCI spellers due to lower signal-to-noise ratio in spontaneous EEG features. Other brain imaging methods, such as semi-invasive electrocorticography (ECoG) and non-invasive functional magnetic resonance imaging (fMRI), have also attracted increasing attention due to their higher spatial discrimination than EEG. Studies using ECoG to decode vowels (Ikeda et al., 2014), vowels and consonants (Pei et al., 2011), and phonemes (Leuthardt et al., 2011); and fMRI to decode words “yes” and “no” (Naci et al., 2013) showed relatively higher decoding performance than EEG studies. Although the performance was still lower than that of reactive BCI spellers, these findings indicate that speech intention can be decoded using brain activity signals if limitations in EEG spatial discrimination can be overcome.

In this study, we demonstrated a method to enhance the utility of EEG in speech intention decoding by using EEG cortical current signals to classify imagined Japanese vowels. Applying a hierarchical Bayesian method (Sato et al., 2004; Yoshioka et al., 2008) that incorporates fMRI activity as a hierarchical prior, spatial discrimination of EEG was improved while preserving its high temporal discrimination. The method is also useable in real-time application since fMRI data need only be acquired one time in advance. The efficacy of this method has already been proven by studies on decoding of motor control (Toda et al., 2011; Yoshimura et al., 2012), visual processing (Shibata et al., 2008), and spatial attention (Morioka et al., 2014). Moreover, since ECoG-based spellers (Leuthardt et al., 2011; Pei et al., 2011; Ikeda et al., 2014) showed relatively higher decoding performance than EEG-based spellers, we hypothesized that EEG cortical current signals would also offer higher decoding performance because EEG cortical current signals are theoretically equivalent to ECoG signals if current dipoles are assigned to the cortical surface. Ten healthy human participants performed covert vowel articulation tasks (i.e., silent production of vowel speech in one's mind, Perrone-Bertolotti et al., 2014), and EEG cortical current signals were estimated using EEG and fMRI data. Classifiers based on sparse logistic regression (SLR) (Yamashita et al., 2008) were trained to discriminate between tasks, and classification accuracies were compared between EEG cortical currents and EEG sensor signals.

## Materials and methods

### Participants

Ten healthy human participants (1 female and 9 males; mean age ± standard deviation: 34.1 ± 9.2) participated in this study. All participants had normal hearing. Written informed consent was obtained from all participants prior to the experiment. The experimental protocol was approved by ethics committees of the National Center of Neurology and Psychiatry and Tokyo Institute of Technology. All participants underwent an fMRI experiment to obtain prior information for EEG cortical current estimation, structural MRI acquisition to create an individual brain model, an EEG experiment, and three-dimensional position measurements of the EEG sensors on the scalp.

### Experimental tasks

**Figure 1:**
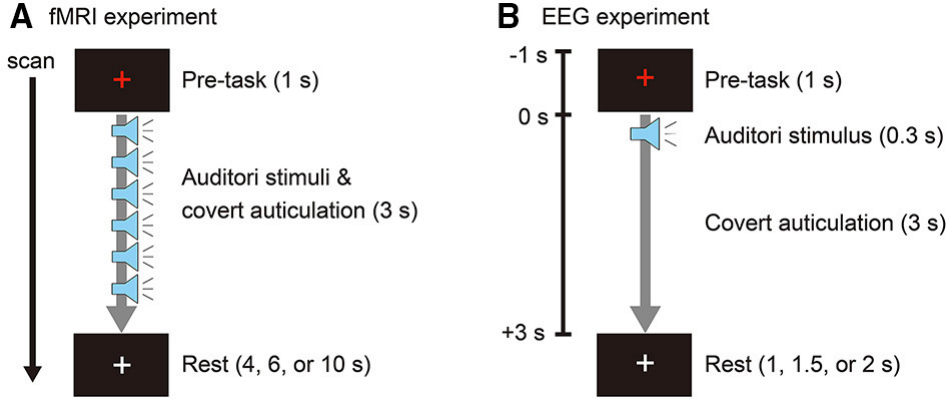
**(A)** Event-related design for the fMRI experiment. One trial consisted of a pre-task (1 s), task (3 s) with six auditory stimuli (0.3 s each), and rest (4, 6, or 10 s). The rest duration was randomly assigned for each trial. The color of the fixation cross was red during the pre-task and task intervals. **(B)** Experimental design for the EEG experiment. One epoch consisted of a pre-task (1 s), task (3 s) with auditory stimulus (0.3 s), and rest (1, 1.5, or 2 s). The rest duration was randomly assigned for each epoch. The color of the fixation cross was red during the pre-task and task intervals.

Participants covertly articulated two Japanese vowels (/a/ and /i/) cued with auditory stimuli. Auditory stimuli were obtained from the Tohoku University - Matsushita Isolated Word Database (Speech Resources Consortium, National Institute of Informatics, Tokyo, Japan) and edited using Wave Editor (Abyss Media Company, Ulyanovsk, Russia) to create stimuli consisting of a single vowel. White noise was created using MATLAB R2012b (The MathWorks, Inc., Natick, MA) and used as a stimulus for a non-imagery (control) task. Stimulus duration was 400 ms, with 300 ms of speech and 100 ms of silence. In the fMRI experiment, auditory stimuli were presented in 3-s durations, with each duration consisting of six repetitions of vowel speech or white noise. Participants covertly repeated the vowel for 3 s when presented with a vowel stimulus, and they refrained from imagery when presented with white noise. We used six repetitions of stimulus and covert speech to obtain higher brain activity signals related to the tasks (Figure 1A). In the EEG experiment, the auditory stimuli were presented only once in the 3-s durations for a single (no-repetition) covert articulation (Figure 1B). In both experiments, to prevent eye and head movement artifacts, a white fixation cross was presented at the center of the experiment screen. The color of the fixation cross changed to red 1 s before each stimulus interval and remained red for 4 s before returning to white.

### fMRI experiment and data acquisition

The fMRI experiment was conducted to identify brain activation areas and their intensities for use as hierarchical priors when estimating cortical currents from EEG (Sato et al., 2004). Participants lay in a supine position on the scanner bed and wore MR compatible headphones (SereneSound, Resonance Technology Inc., Northridge, CA). Auditory stimuli were provided at a sound pressure level of 100 dB. The fixation cross was projected on a screen and viewed through a mirror attached to the MRI head coil. To confirm task execution, participants pressed a response button with their right hand when they heard the auditory stimuli. Using an event-related design, one trial was comprised of a pre-task period (1 s), a task period (3 s), and a rest period (4, 6, or 10 s, pseudo-randomly permuted over three consecutive trials; Figure 1A). Participants performed three runs, with each run consisting of 10 trials for each task (/a/, /i/, and no-imagery). Tasks were ordered in pseudo-random permutations over three consecutive trials. The experiment program was created using Presentation version 16.3 (Neurobehavioral Systems, Inc., Berkeley, CA).

A 3 T Magnetom Trio MRI scanner equipped with an 8-channel array coil (Siemens, Erlangen, Germany) was used for the fMRI experiment. Functional data were acquired with a T2^*^-weighted gradient-echo, echo planar imaging sequence using the following parameters: repetition time (TR) = 3 s; echo time (TE) = 30 ms; flip angle (FA) = 90°; field of view (FOV) = 192 × 192 mm; matrix size = 64 × 64; 43 slices; slice thickness = 3 mm; 118 volumes.

After the fMRI experiment, two types of 3D anatomical images, a sagittal image and an axial image, were acquired using T1-weighted magnetization prepared rapid gradient echo sequences (for sagittal scans: *TR* = 2 s; *TE* = 4.38 ms; *FA* = 8°; *FOV* = 256 × 256 mm; matrix size = 256 × 256; 224 slices; slice thickness = 1 mm; for axial scans: *TR* = 2 s; *TE* = 4.38 ms; *FA* = 8°; FOV = 192 × 192 mm; matrix size = 192 × 192; 160 slices; slice thickness = 1 mm). The sagittal image covered the whole head, including the face, specifically for use in constructing a polygon model of the cortical surface.

### EEG experiment and data acquisition

Participants were seated in a sound-attenuated chamber (AMC-3515, O'HARA & CO., LTD. Tokyo, Japan) and wore earphones (Image S4i, Klipsch Group, Inc., Indianapolis, IN) providing auditory stimuli at a sound pressure level of 70 dB. Tasks were presented in pseudo-random permutations with randomly assigned interstimulus intervals of 2, 2.5, or 3 s. Participants performed 10 sessions, with each session consisting of 5 trials per task. Eye blinking was allowed only during the interval period when a white fixation cross was presented (Figure 1B).

**Figure 2:**
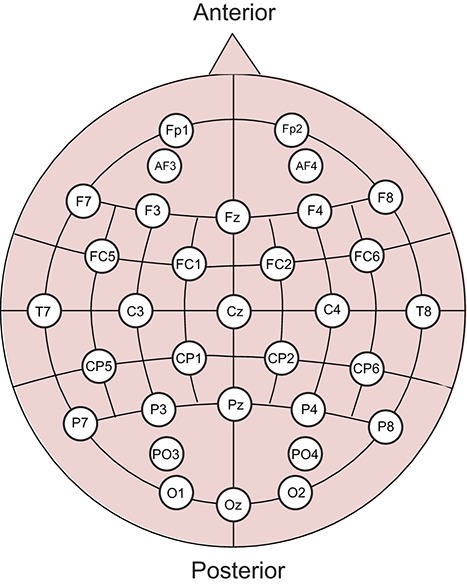
**Sensor positions in the EEG experiment**. Thirty-two electrodes were used based on the extended international 10–20 system.

EEG signals were recorded using a g.USBamp amplifier/digitizer system and 32 g.LADYbird active sensors (g.tec medical engineering, Graz, Austria). The resolution and range of the amplifier were 30 nV and ±250 mV, respectively. The sensors were positioned according to the extended international 10–20 system (Figure 2), and the average from both earlobes was used as reference. The scalp was cleaned with 70% ethanol before filling the electrode gaps with conducting gel. Signals were acquired at a sampling rate of 256 Hz and band-pass filtered from 0.5 to 100 Hz using an experiment program created in MATLAB 2012b.

After the EEG experiment, coordinate positions of EEG sensors were measured using a Polaris Spectra optical tracking system (Northern Digital Inc., Ontario, Canada). The nasion, left pre-auricular point and right pre-auricular point were also measured as reference points. These reference points were used to co-register the EEG sensor positions to voxel coordinates of the T1-weighted sagittal image.

### EEG cortical current estimation using a hierarchical bayesian methods

EEG cortical currents were estimated using Variational Bayesian Multimodal Encephalography (VBMEG) toolbox (ATR Neural Information Analysis Laboratories, Japan; http://vbmeg.atr.jp/?lang=en) running on MATLAB R2012b, unless otherwise specified. All VBMEG processes were conducted in accordance with standard procedures described in the toolbox documentation.

#### Cortical surface model and three-layer model

We constructed a cortical surface model comprised of current dipoles equidistantly distributed on and perpendicular to the cortical surface, which consisted of sulci and gyri. We also constructed a three-layer model consisting of boundary information for scalp, skull, and cerebrospinal fluid. The T1-weighted sagittal image was used for creating the two models. A bias corrected image and a gray-matter volumetric image of the sagittal image were obtained using the “Segment” function in SPM8 (Wellcome Department of Cognitive Neurology, UK; available at http://www.fil.ion.ucl.ac.uk/spm). The bias corrected image was used for creating a polygon model of the cortical surface in FreeSurfer (Martinos Center software, http://surfer.nmr.mgh.harvard.edu/), followed by the cortical surface model. The gray-matter volumetric image was used in conjunction with the output files of FreeSurfer to create the three-layer model.

#### fMRI data processing

fMRI data were analyzed with SPM8 and VBMEG. Slice-timing correction was performed on all echo planar images (EPIs), followed by spatial realignment to the mean image of all EPIs. The T1-weighted axial image was co-registered to the T1-weighted bias-corrected sagittal image before all realigned EPIs were co-registered to the T1-weighted axial image. Then, all EPIs were spatially normalized to the Montreal Neurological Institute (MNI) (Montreal, Quebec, Canada) reference brain via the T1-weighted bias-corrected sagittal image, and spatially smoothed with a Gaussian kernel of 8 mm full-width at half-maximum.

In SPM first-level analysis, for each participant, we calculated *t*-values for two contrasts task /a/ > task /i/ and task /i/ > task /a/ using *p*-value thresholds for uncorrected multiple comparisons of 0.001, 0.005, 0.01, 0.05, and 0.1 (termed “single-activation”). All of the *t*-values were then used to define two kinds of priors, area (the number of cortical vertices to be estimated) and activity (affecting current amplitude of each vertex) in VBMEG. Using VBMEG, the *t*-values of the respective contrast images were inverse-normalized into individual participant's space and co-registered to the cortical surface model as area and activity priors. Merging priors across the two contrasts at each *p*-value threshold, we created final area and activity priors for EEG cortical current estimation for each participant.

We also created other sets of area and activity priors for each participant using results from SPM group (second-level) analyses. A one-sample *t*-test was performed for the contrast between task /a/ and task /i/ (*p* = 0.01 uncorrected). Group analyses were performed for 11 conditions. One analysis used all participants' images (“Group-activation”), and the remaining 10 analyses used 9 participants' images, changing combinations in a leave-one-participant-out manner (“LOOgroup-activation”). The *t*-value images of the group-activations and LOOgroup-activations were inverse normalized into individual participant's space to create area and activity priors of each participant.

#### EEG data processing

EEG sensor signals were low-pass filtered at a cutoff frequency of 45 Hz, and 50 epochs per task were extracted in reference to auditory stimulus onsets. Each epoch had a duration of 4 s, 1 s of pre-onset and 3 s of post-onset. Coordinate positions of EEG sensors were co-registered to the T1-weighted bias-corrected sagittal image using positioning software supplied with VBMEG.

#### Inverse filter and cortical current time course estimation

To design an inverse filter in VBMEG, several parameters, including two hyper-parameters, must be defined through a current variance estimation step. We determined the best hyper-parameters using a nested cross-validation method described in Section Vowel Classification Analysis using Sparse Logistic Regression. The other parameters were defined as follows: analysis time range = −0.5–3 s; time window size = 0.5 s; shift size = 0.25 s; dipole reduction ratio = 0.2. We then used the inverse filter to calculate cortical current time courses for trial data at 0.5–3 s. Parameters differed for area and activity analyses (described in Section Vowel Classification Analysis using Sparse Logistic Regression). The two hyper-parameters, a prior magnification parameter (*m*_0_) and a prior reliability (confidence) parameter (γ_0_), were selected from *m*_0_∈{10, 100, 1000} and γ_0_∈{1, 10, 100}, respectively. Both parameters constrain influence of fMRI data on current variance estimation (Sato et al., 2004). A larger magnification parameter *m*_0_ leads to a larger dipole current amplitude for a given fMRI activation. A larger reliability parameter γ_0_ requires a more sharply peaked fMRI activation in the variance distribution. For both, high values signify that brain activity during the fMRI experiment was the same as that during the EEG experiment. We chose their values based on a method by Morioka et al. (2014). However, we did not select high values because our fMRI and EEG data were not recorded simultaneously but on different days using slightly different experimental protocols.

### Vowel classification analysis using sparse logistic regression

We applied sparse logistic regression (SLR) (Yamashita et al., 2008) for vowel classification, which can train high-dimensional classifiers without need for advance dimension reduction. Three-class classifiers for /a/, /i/, and no-imagery (control) were trained based on sparse multinomial logistic regression (SMLR) using SLR Toolbox version 1.2.1 alpha (Advanced Telecommunications Research Institute International, Japan; http://www.cns.atr.jp/~oyamashi/SLR_WEB.html). SMLR trained three classifiers for the individual three tasks, and it chose the class with the highest probability using test data.

For both EEG sensor and cortical current signals, the signals were passed through an 8-point moving average filter, and data from 1.0 to 2.0 s of post-onset were used for analysis to avoid influence by auditory or event-related evoked potentials, such as N1 and P300, and also by motor-related potentials evoked by pressing the response button. Classification accuracies for EEG cortical current signals were evaluated in a nested cross-validation manner to observe the influence of the hyper-parameters. For EEG sensor signals, nested cross-validation was not applied because hyper-parameter tuning was not applicable. Instead, 5 × 10-fold cross validation was used, which repeats a 10-fold cross validation 5 times, randomizing the trials in a fold for each iteration. This repeated cross-validation method was applied to obtain more generalized classification accuracies.

**Figure 3:**
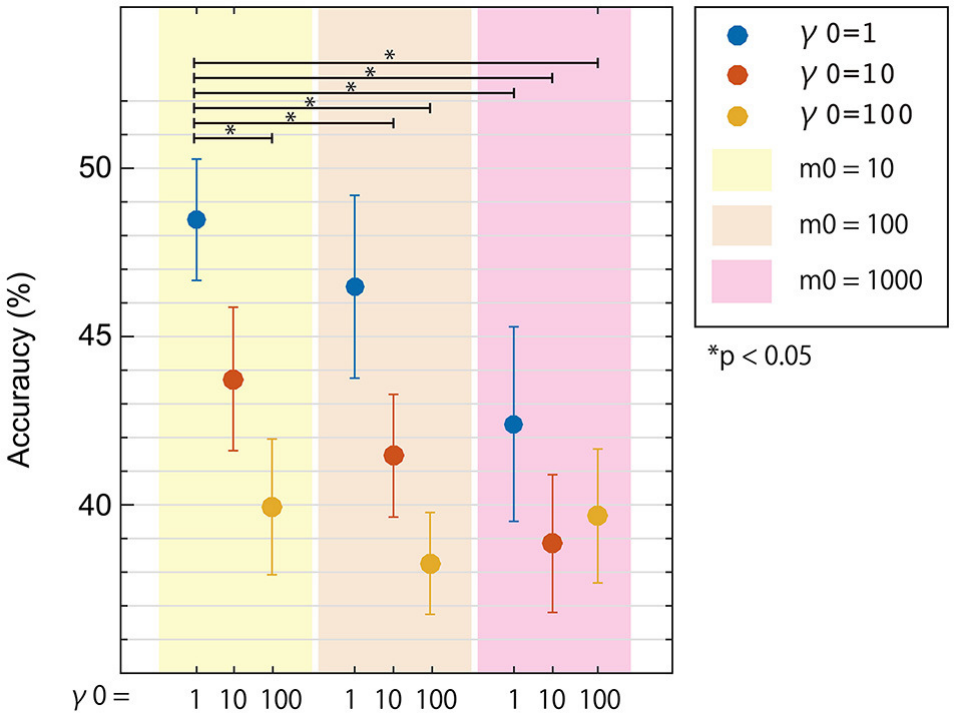
**Three-class classification accuracies of EEG cortical current signals obtained from inverse filters with different hyper-parameters**. Results using a prior magnification parameter *m*_0_ = 10, 100, and 1000 are denoted as yellow, cream, and pink areas, respectively. Results using prior reliability parameters γ_0_ = 1, 10, and 100 are denoted as blue, red, and yellow dots, respectively. All conditions used the same fMRI area/activity priors from Unc001. Error bars denote standard error. Statistical differences were calculated using non-parametric permutation tests.

To see the influence of the hyper-parameters on classification accuracy, we used fixed area/activity priors of *p* = 0.01 (single-activation) as shown in Figure 3. The 50 trials for each task were randomly divided into 10 groups, with 5 trials each. Eight groups were used to train a classifier using one of nine hyper-parameter pairs (where *m*_0_∈{10, 100, 1000} and γ_0_∈{1, 10, 100}). One of the remaining groups was used as validation data to calculate classification accuracies for the pair of hyper-parameters. The last remaining group was reserved as test data to be used after the best hyper-parameters were found. The process was repeated 9 times for each permutation of training and validation data, and mean classification accuracies were calculated for each validation. This was repeated for all parameters pairs, and mean accuracies across participants were plotted in Figure 3.

Next, the pair of hyper-parameters with the highest accuracy was selected, and a new classifier was trained using both the training and validation data (9 groups), and net classification accuracy was calculated using the test data reserved in the first step. This step was performed to compare accuracies among different area/activity priors. The process was repeated 10 times per pair of fMRI area/activity priors, changing the group used as test data in a leave-one-group-out manner. The entire nested cross-validation process was further repeated 5 times, randomizing the trials in each of the 10 groups.

We calculated accuracies for the following 8 fMRI prior pairs (Table 1): 5 single-activations (uncorrected *p* = 0.001, 0.005, 0.01, 0.05, and 0.1), Group-activation (Group, uncorrected *p* = 0.01), LOOgroup-activation (LOOgroup, uncorrected *p* = 0.01), and LOOgroup with data labels randomized during classifier training (RandLOOgroup, uncorrected *p* = 0.01). We included the RandLOOgroup condition to determine if accuracies increased simply due to increased data dimensionality.

**Table 1:** **List of conditions used for designing inverse filters for EEG cortical currents**.

**Condition**	***P*****-value threshold**		**Hyper-parameters**		**The number of vertices**	**Accuracy (%)**
	**For area**	**For activity**	***m*0**	**γ0**		
Unc0001	0.001	0.001	10, 100, 1000	1, 10, 100		
Unc0005	0.005	0.005	10, 100, 1000	1, 10, 100		
Unc001	0.01	0.01	10, 100, 1000	1, 10, 100		
Unc005	0.05	0.05	10, 100, 1000	1, 10, 100		
Unc01	0.1	0.1	10, 100, 1000	1, 10, 100		
Group	0.01	0.01	10	1		
LOOgroup	0.01	0.01	10	1		
RandLOOgroup	0.01	0.01	10	1		
**Bestcond**						
P1	0.01	0.001	10	10	229	63.3
P2	0.01	0.01	10	10	47	62.7
P3	0.005	0.005	10	100	13	50.0
P4	0.01	0.001	100	100	83	49.3
P5	0.01	0.01	100	10	260	46.7
P6	0.05	0.05	10	100	335	66.0
P7	0.001	0.001	10	1	130	54.0
P8	0.01	0.01	10	10	31	56.0
P9	0.01	0.01	10	10	206	59.3
P10	0.1	0.01	10	1	663	77.3

*In Unc0001, Unc0005, Unc001, Unc005, and Unc01 conditions, the same p-values for area and activity were used from individual participant SPM results, and 9 inverse filters were calculated for each condition using all combinations of the two hyper-parameters. In Group, LOOgroup, and RandLOOgroup conditions, p-value thresholds and hyper-parameters were the same because data used in SPM second-level analysis differed for Group and LOOgroup, and RandLOOgroup data used the same data as LOOgroup with randomized labeling. In Bestcond condition, parameters that showed the highest accuracies were listed along with the number of vertices obtained from the estimation*.

**Figure 4:**
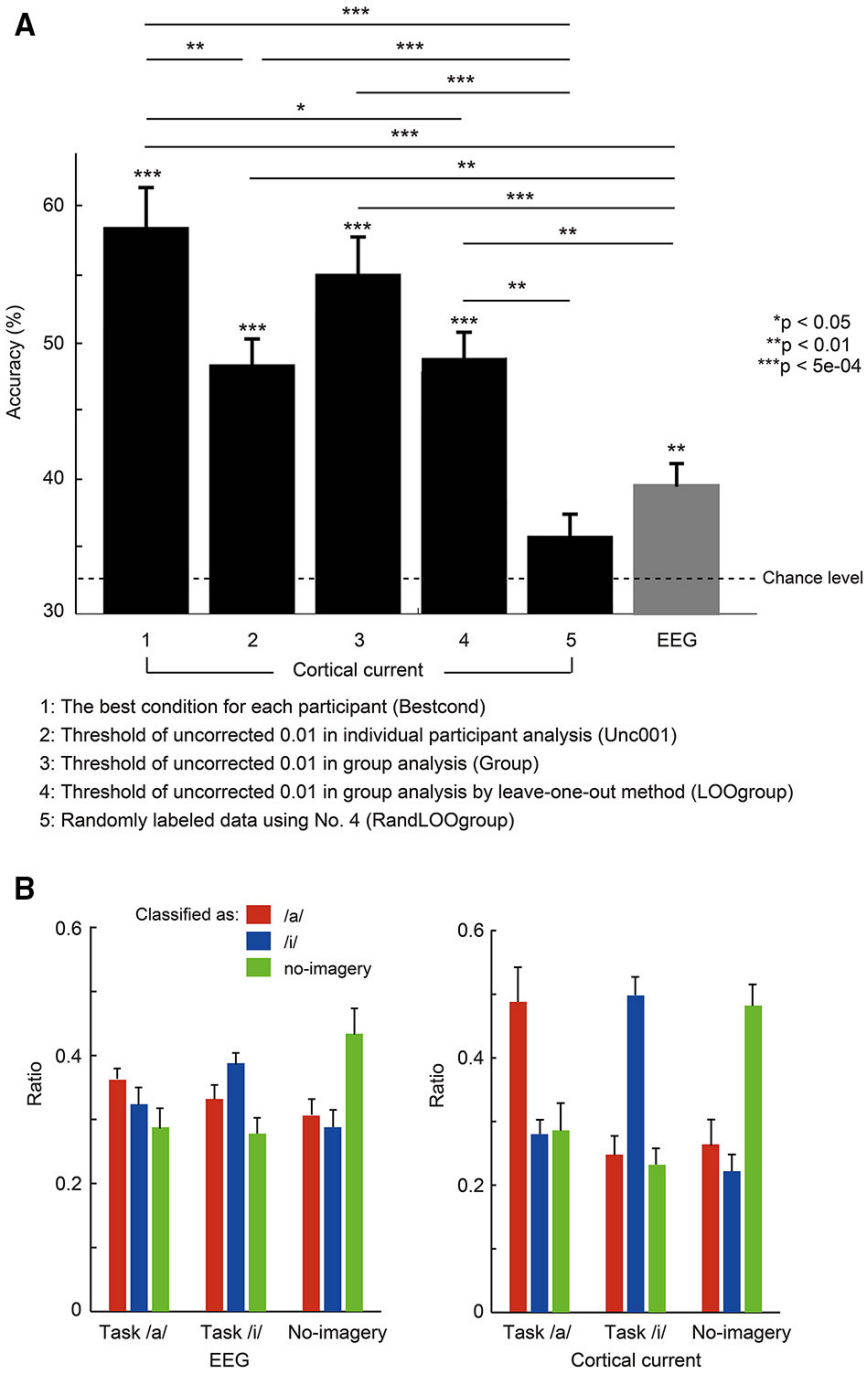
**(A)** Mean accuracies averaged across 10 participants for the three-class classification using EEG sensor and cortical current data. Error bars denote standard error. The dotted line denotes chance level of 33.3%. ^*^p < 0.05, ^**^p < 0.01, ^***^p < 5e-04 usnig non-parametric permutation tests. Parameters used for each condition (No. 1–No. 5) are shown in Table 1. **(B)** Comparison of classification output ratio for each task resulting from EEG sensor (*left*) and cortical current (*right*) classification. Using a probability map from each classification analysis, the number of times that marked the highest probability was counted for each task. Then the ratio of that number to the total number of cross-validations was calculated and averaged across participants. For each task, red bars represent the ratio classified as vowel /a/, blue bars as vowel /i/, and green bars as no-imagery.

In Figure 4A, we compared mean classification accuracies across participants using the net accuracies among the prior pairs. Note that we did not show all mean accuracies for single-activations but rather mean calculated using the best accuracies among the results of the single activations (termed “Bestcond”). Conditions that gave the best accuracy for each participant are shown in Table 1. Statistical analyses to assess significant difference between them were performed using non-parametric permutation tests (Nichols and Holmes, 2002; Stelzer et al., 2013; Yoshimura et al., 2014), which compare the mean classification accuracy to other mean accuracies that were calculated repeatedly using randomly permuted class-labels and calculated *p*-values. The comparison was repeated 10,000 times using pseudo-randomized labels. Therefore, the labeling with the highest overall difference would have a *p*-value of 1/10,000 = 1.00e-04.

### Evaluation of EEG cortical current estimation for contribution to vowel classification

**Figure 5:**
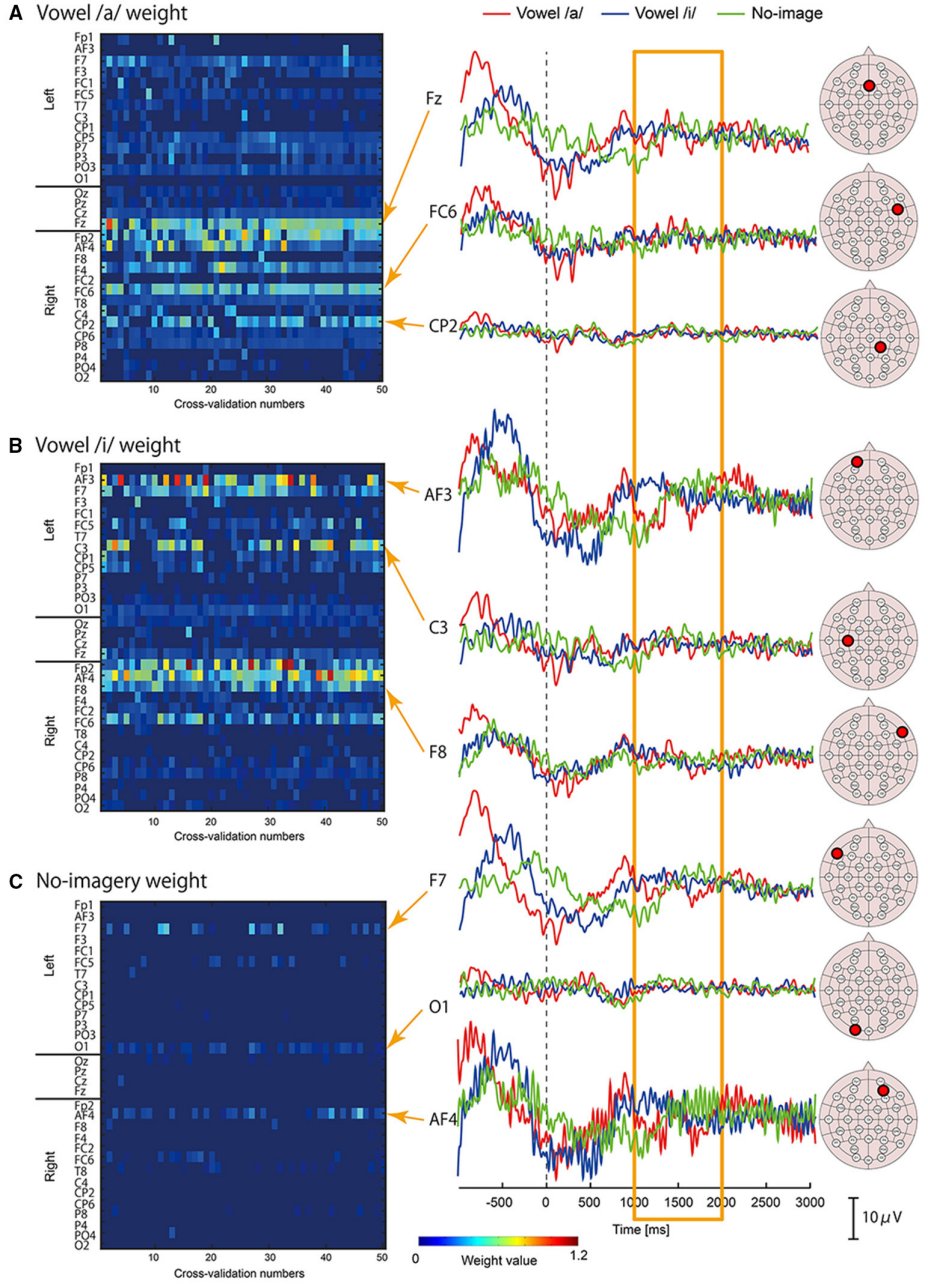
**Examples of EEG sensor signals in a participant who showed the highest accuracy**. Left panel: Color maps of mean weight values of all sensors for each cross-validation analysis calculated by classifier for vowel /a/ **(A)**, vowel /i/ **(B)**, and no-imagery **(C)**. Right panel: Frequently selected sensors with high mean weight values (FSHV-sensors) were selected from the color maps and mean time series signals across trials were plotted. Red lines represent signals during vowel /a/ task, blue lines represent vowel /i/ task, and green lines represent no-imagery task. Signals from 1 to 2 s after the auditory stimuli (orange box) were used for classification analyses. EEG sensor positions of the FSHV-sensors are shown beside each signal plot.

**Figure 6:**
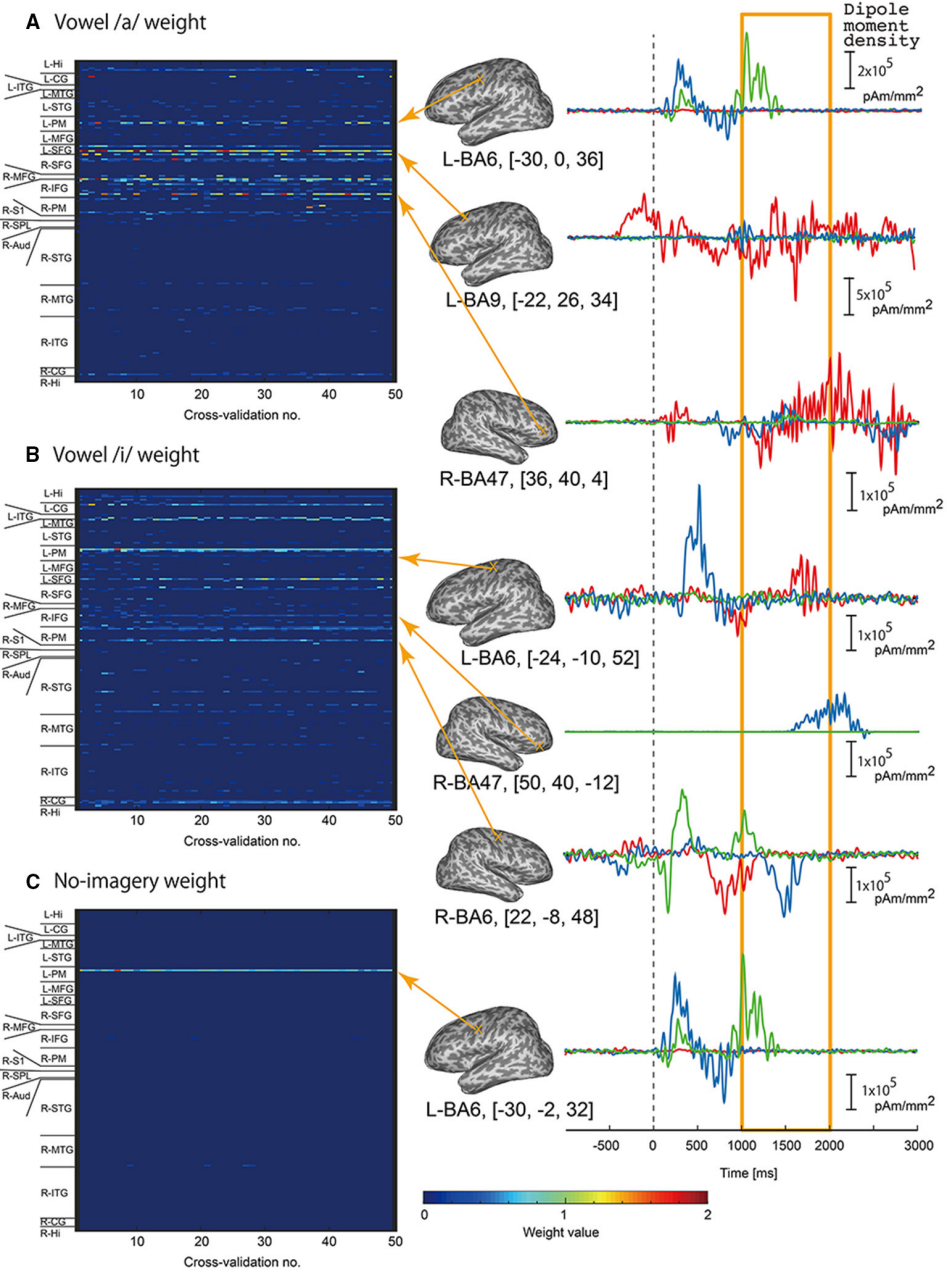
**Examples of EEG cortical current signals in the same participant in Figure 5**. Left panel: Color maps of mean weight values of all vertices sorted according to brain areas for each cross-validation analysis calculated by classifier for vowel /a/ **(A)**, vowel /i/ **(B)**, and no-imagery **(C)**. Right panel: Frequently selected vertices with high mean weight values (FSHV-vertices) were selected from the color maps, and mean time series signals across trials were plotted. Red lines represent signals during vowel /a/ task, blue lines represent vowel /i/ task, and green line represent no-imagery task. Signals from 1 to 2 s after the auditory stimuli (orange box) were used for classification analyses. Positions of the FSHV-vertices and their MNI standard coordinates are shown beside each signal plot. MNI standard coordinates of the current vertices were calculated using the normalization matrix obtained from SPM analysis to determine anatomical locations of the current vertices.

We examined which cortical vertices or EEG sensors contributed to vowel classification by analyzing weight values of the three-class classifiers. For each classifier of /a/, /i/, and no-imagery, weight values were normalized by the maximum weight value in each cross validation, and mean weight values of time point features (32 points per vertex) for each vertex or EEG sensor were then calculated. The mean weight values for all of the vertices or EEG sensors were plotted as a colored map for all cross-validation times (Figure 5 for EEG sensors, Figure 6 for cortical vertices). For some vertices and EEG sensors that were frequently selected by the cross-validation analysis, we compared differences in time course signals during the three tasks. Mean time course signals were calculated across trials and plotted for EEG sensors (Figure 5) and EEG cortical currents (Figure 6).

**Figure 7:**
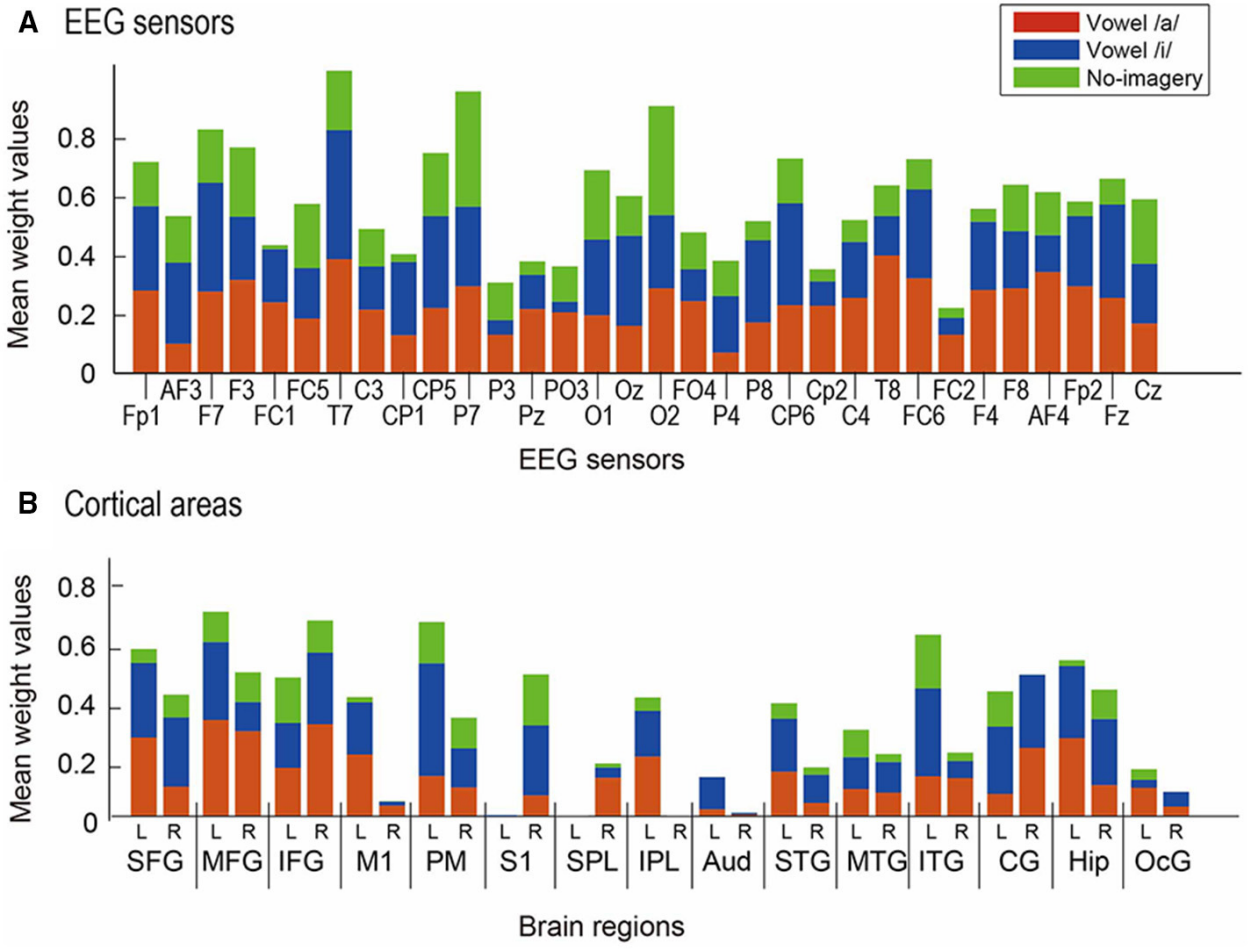
**Mean normalized weight values across participants**. Red bars represent results from weight for vowel /a/, blue bars for vowel /i/, and green bars for no-imagery. **(A)** Values for 32 EEG sensors were compared. **(B)** Values for 30 ROIs were compared: left (L) and right (R) hemisphere of SFG, MFG, IFG, M1, PM, S1, SPL, IPL, Aud, STG, MTG, ITG, CG, Hip, and OcG.

For cortical vertices, we further calculated mean weight value of each classifier across participants for 30 anatomical regions to find the most contributive anatomical area for the classification (Figure 7). Since MNI coordinate positions of cortical vertices were stored when constructing the cortical surface model in VBMEG, we were able to examine the anatomical regions corresponding to the locations of the cortical vertices using SPM Anatomy toolbox (Version 1.7) (Eickhoff et al., 2005) (http://www.fz-juelich.de/inm/inm-1/DE/Forschung/_docs/SPMAnatomy Toolbox/SPMAnatomyToolbox_node.html). We defined the following 30 anatomical regions of interest (ROIs) for the contribution analysis: left and right hemispheric superior frontal gyrus (L/R-SFG), middle frontal gyrus (L/R-MFG), inferior frontal gyrus (L/R-IFG), primary motor cortex (L/R-M1), premotor cortex (L/R-PM), primary somatosensory cortex (L/R-S1), superior parietal lobule (L/R-SPL), inferior parietal lobule (L/R-IPL), primary auditory cortex (L/R-Aud), superior temporal gyrus (L/R-STG), middle temporal gyrus (L/R-MTG), inferior temporal gyrus (L/R-ITG), cingulate gyrus (L/R-CG), hippocampus (L/R-Hip), and occipital gyrus (L/R-OcG).

**Figure 8:**
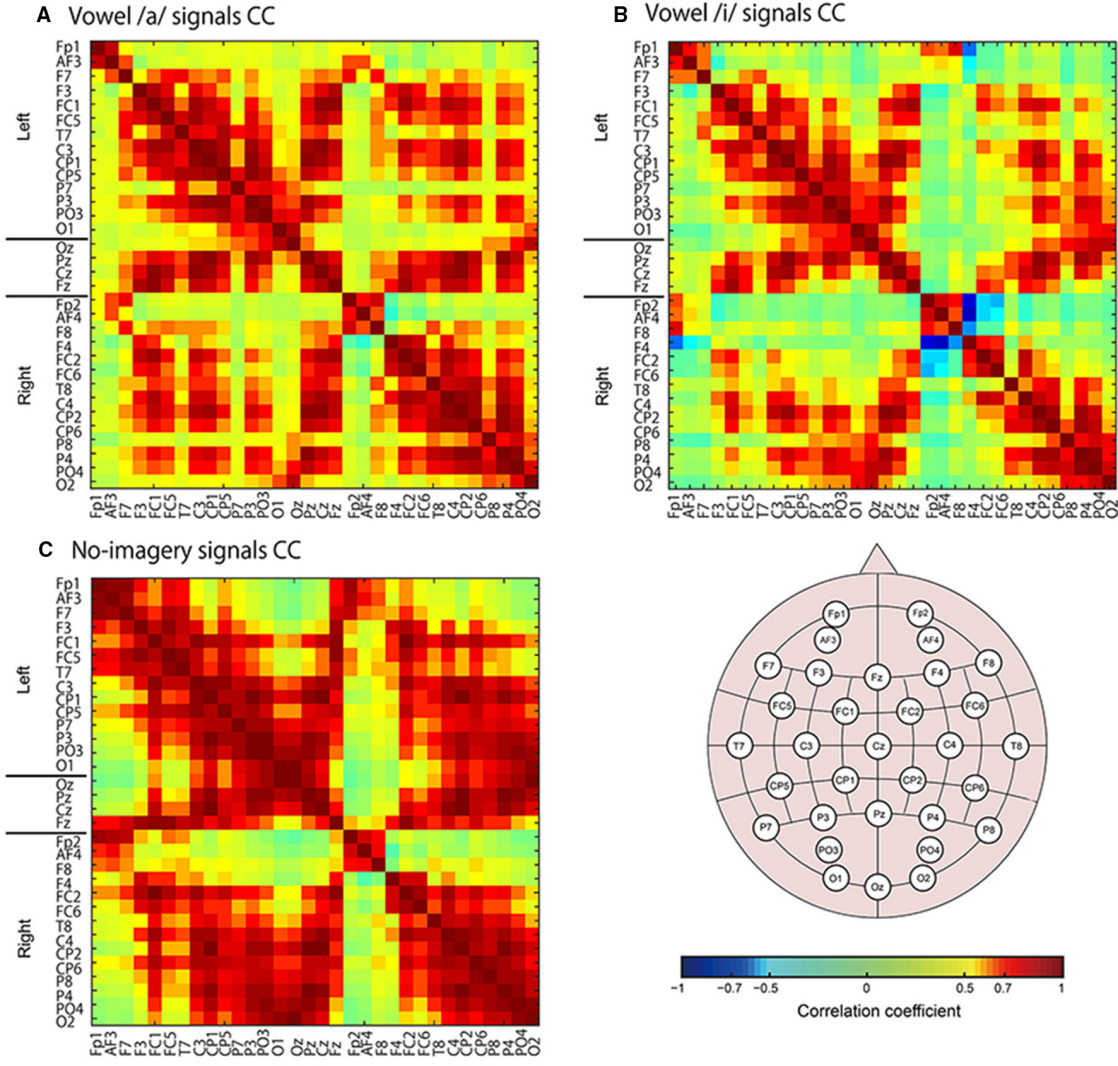
**Color maps of correlation coefficient (CC) values for EEG sensor signals**. Sensors names are listed along with the horizontal and vertical axes. CC values were calculated for all tasks signals: vowel /a/ **(A)**, vowel /i/ **(B)**, and no-imagery **(C)**.

**Figure 9:**
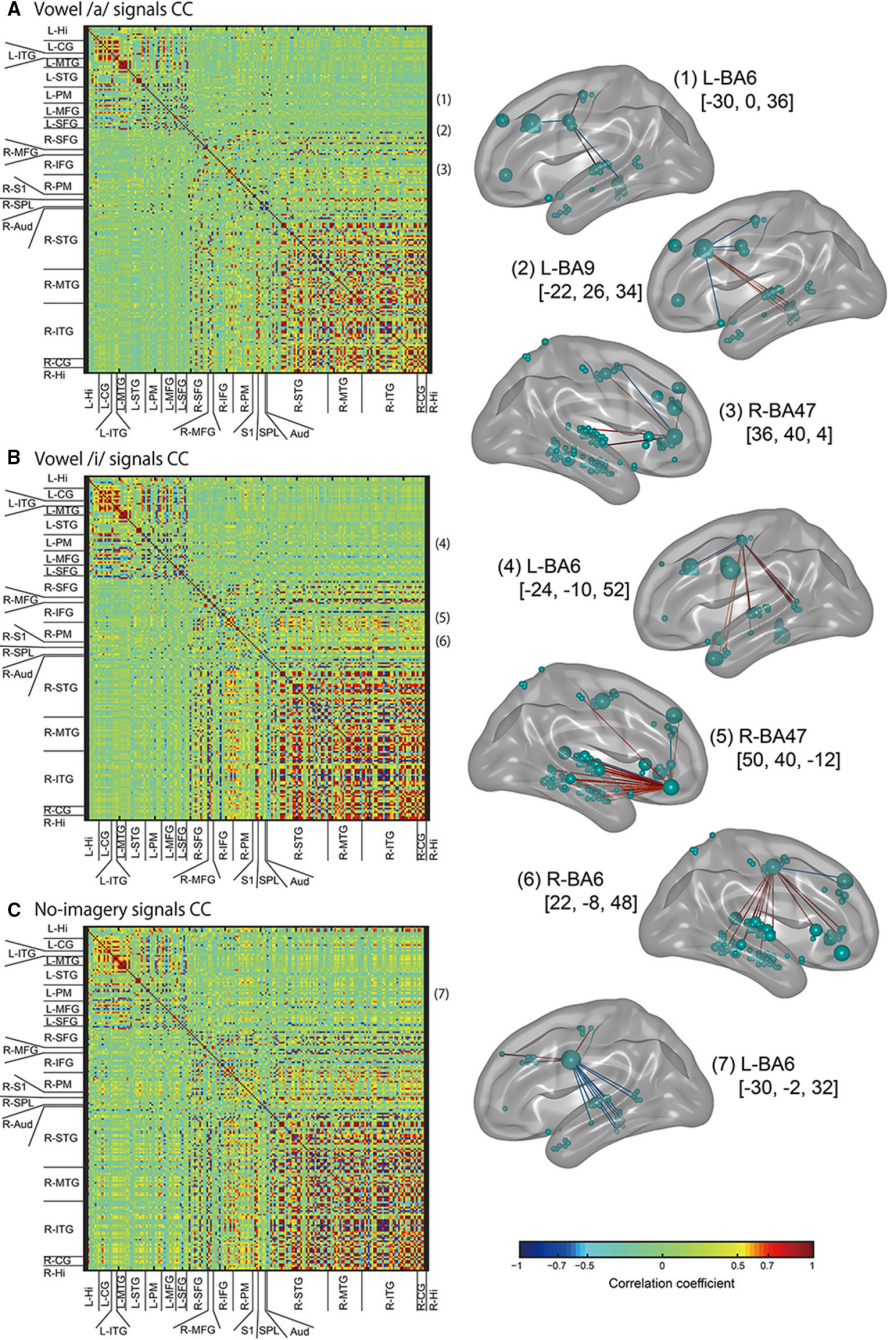
**Left panel: Color maps of correlation coefficient (CC) values for EEG cortical current signals**. ROIs names that cortical vertices were included in are listed along with the horizontal and vertical axes. CC values were calculated for all tasks signals: vowel /a/ **(A)**, vowel /i/ **(B)**, and no-imagery **(C)**. Right panel: The FSHV-vertices defined in Figure 6 [(1–7) in this figure) were set as seeds, and positive correlations above 0.6 (red lines) and negative correlations below–0.6 (blue lines) were visualized with BrainNet Viewer (Xia et al., 2013) (http://www.nitrc.org/projects/bnv/). Green balls on the brain maps denote vertices, with their sizes representing mean weight values, and names of the brain areas in Brodmann style and MNI coordinates of the seeds are written besides the brain maps.

Moreover, we calculated coefficient of correlations for all vertex pairs and all EEG sensor pairs to find vertices or sensors that highly correlate with frequently selected vertices or sensors (Figures 8, 9).

## Results

### Comparison of hyper-parameters for EEG cortical current estimation

In Figure 3, three-class classification accuracies from the nested cross-validation analysis were plotted for the 9 pairs of hyper-parameters. All mean accuracies were higher than chance level (33.3%) and differed according to the hyper-parameters used. Also, the best pair of hyper-parameters was not always the same in each cross-validation step. As expected, we obtained higher accuracies using lower hyper-parameter values. Since both hyper-parameters represented similarity between fMRI and EEG activity patterns, this result is reasonable because we recorded EEG and fMRI data on different days using slightly different experimental protocol. We used the hyper-parameters that provided the highest accuracy, *m*_0_ = 10 and γ_0_ = 1, in the next analysis (Section Comparison between Classification Accuracies for EEG Sensors and Cortical Currents) comparing accuracies for different fMRI activity priors.

### Comparison between classification accuracies for EEG sensors and cortical currents

Figure 4A compares three-class classification accuracies (/a/, /i/, and no-imagery) of EEG sensor and EEG cortical current signals estimated using different fMRI area/activity priors. All values are mean accuracies across participants. All accuracies except randomly labeled data (No. 5) were significantly higher than chance level (Bestcond: 58.5 ± 2.93%, *p* = 2.00e-04; Unc001: 48.5 ± 1.80%, *p* = 1.00e-04; Group: 55.0 ± 2.55%, *p* = 1.00e-04; LOOgroup: 48.8 ± 1.90%, *p* = 1.00e-04; RandLOOgroup: 35.7 ± 1.43%, *p* = 0.08; EEG: 39.5 ± 1.62%, *p* = 7.00e-04; using permutation tests). Furthermore, all EEG cortical currents data except randomly labeled data showed significantly higher accuracies than EEG data (Bestcond: *p* = 1.00e-04; Unc001: *p* = 0.0018; Group: *p* = 4.00e-04; LOOgroup: *p* = 0.0024; RandLOOgroup: *p* = 0.11; using permutation tests). Since the randomly labeled data (RandLOOgroup) showed nearly chance-level accuracy, accuracy increases using EEG cortical currents likely could not be attributed to increased data dimensionality but rather to meaningful information being extracted by EEG cortical current estimation.

Comparing accuracies for different fMRI priors (No. 1–No. 4), Bestcond (58.5%) showed significantly higher accuracy than all other conditions except the Group condition. Interestingly, the Group condition (55.0%) showed relatively higher accuracy than Unc001 even though activation of the Group condition was obtained using all participants in Unc001 (Bestcond vs. Group: *p* = 0.39; Unc001 vs. Group: *p* = 0.050; Unc001 vs. LOOgroup: *p* = 0.91; Unc001 vs. Bestcond: *p* = 0.0076; Group vs. LOOgroup: *p* = 0.065; LOOgroup vs. Bestcond: *p* = 0.011; using permutation tests).

Classification ratios (Figure 4B) further revealed that there was no disproportion in true-positive classifications among tasks. Specifically, only in EEG cortical currents were true-positives significantly more frequent than false-positives and false-negatives, and no significant difference in true-positives was observed between tasks [cortical currents: *F*_(8, 81)_ = 10.8, *p* = 3.11e-10; EEG: *F*_(8, 81)_ = 3.8, *p* = 0.001; by one-way ANOVA). Therefore, classification accuracy (Figure 4A) was not the result of prominently high accuracy in any single task, and there was no disproportion in accuracies for the three tasks.

### Contribution of brain regions to classification of covert vowel articulation

Left panels of Figures 5A–C, 6A–C show weight analysis results of EEG and cortical current classifiers, respectively, in a participant who showed the highest accuracy among the participants. For both EEG sensor and cortical current signals, the same sensors or vertices tended to be selected throughout the cross-validation. These tendencies were observed for all participants, even though locations of frequently selected sensors or vertices differed between participants.

When calculating mean weight values of the 30 ROIs across participants (Figure 7), L-SFG, L-MFG, R-IFG, L-PM, L-ITG, and L-Hip showed significantly higher values than R-M1, R-PM, L-S1, LR-SPLs, R-IPL, LR-Auds, R-STG, LR-MTGs, R-ITG, and LR-OcGs [*p* < 0.05 in multi-comparison analysis after a three-way ANOVA (main effect of participant: *F*_(9)_ = 1.63, *p* = 0.17; main effect of ROI: *F*_(29)_ = 3.24, *p* = 0; main effect of task: *F*_(2)_ = 12.8, *p* = 0; interaction between participant and area: *F*_(261)_ = 1.48, *p* = 0.0001; interaction between area and task: *F*_(58)_ = 0.94, *p* = 0.60; multi-comparison results showed no-significance between participants)].

Mean weight values of EEG sensors across participants (Figure 7B) revealed that T7, P7, and O2 showed significantly higher values than the other sensors [*p* < 0.05 in multi-comparison analysis after a three-way ANOVA (main effect of participant: *F*_(9)_ = 0.28, *p* = 0.98; main effect of sensor: *F*_(31)_ = 1.81, *p* = 0.005; main effect of task: *F*_(2)_ = 12.4, *p* = 0; interaction between participant and area: *F*_(279)_ = 1.50, *p* = 0; interaction between area and task: *F*_(62)_ = 1.05, *p* = 0.37; multi-comparison results showed no significance between participants)].

To see the difference in time series signals between tasks for the EEG sensors and vertices that were frequently selected and assigned high weight values (termed “FSHV-sensors” and “FSHV-vertices”, respectively), we compared mean time series signals across trials for the three tasks in the same participant (orange boxes in the right panels of Figures 5A–C, 6A–C. FSHV-sensors tended to be located in the right frontal area (Fz, Fp2, F4, FC6) for vowel /a/ classifiers, in the bilateral frontal area (AF3, F7, C3, Fp2, AF4, F8, and FC6) for vowel /i/ classifiers, in the bilateral frontal and the left occipital areas (F7, AF4, and O1) for no-imagery classifiers in case of the participant. Only slight differences were observed among their signals even though the sensors positions were apart from each other. FSHV-vertices were located in bilateral frontal areas [L-SFG (BA9), L-PM (BA6), and R-IFG (BA47)] for vowel /a/ classifiers, in left temporal and bilateral frontal areas [L-MTG (BA21), L-PM (BA6), L-SFG (BA9), R-IFG (BA47), R-PM (BA6)] for vowel /i/ classifiers, and in left frontal area [L-PM (BA6)] for no-imagery classifiers. The most notable difference with the EEG results was that signal patterns during imagery (orange box in the right panels of Figures 6A–C) were quite different for each task and location.

Next we calculated correlation coefficients (CC) for all EEG sensor- or vertex- pairs of signals to identify neural signaling related to language processes. For EEG sensors (Figure 8), as expected, many pairs of EEG sensor signals showed high CC values, and high correlations were particularly observed between sensors located close to each other, even if located contra-laterally. For EEG cortical currents (Figure 9, left panels), high correlations were limited mainly to vertices located in the same hemisphere. Furthermore, when we drew connectivity maps using the FSHV-vertices as seeds and lines connecting to vertices with absolute CC values of more than 0.6, we found that the FSHV-vertices had positive or negative correlations with vertices located in Brodmann areas (BA) 6, 9, 20, 21, 22, 38, 46, and 47 (Figure 9, right panels).

## Discussion

The aim of this study was to classify brain activity associated with covert vowel articulation. Results demonstrated that using EEG cortical current signals provided significantly higher classification accuracy than that using EEG sensor signals. Accuracy using cortical current signals was also comparable to those of existing studies using semi-invasive ECoG (Leuthardt et al., 2011; Pei et al., 2011; Ikeda et al., 2014). These results seem to be attributed to enhancement in spatial discrimination of EEG using cortical current estimation, since signal patterns of cortical currents were found to differ greatly from each other (Figures 6A–C). The enhancement in spatial discrimination further provided the possibility of findings on neural processing of vowel articulation (Figure 9). Although, EEG cortical current estimation employs fMRI data as hierarchical priors, real-time fMRI data acquisition is not required since the hierarchical priors are used for inverse filter design, and the inverse filter is calculated in advance using pre-recorded fMRI and EEG data. Therefore, EEG cortical current signals are applicable to real-time interfaces. To our knowledge, this is the first study to demonstrate usability for EEG cortical currents in BCI spellers, as well as the contributive anatomical areas and their functional connectivities for covert vowel articulation.

### Brain regions contributive to covert vowel articulation revealed by EEG cortical current signals

Our EEG cortical current results (Figure 7) showed that left MFG (BA46), right IFG (BA47), left PM (BA6), and left ITG (BA20) were highly contributive to the classification of vowels /a/ and /i/ and no-imagery. Though the neural circuit for covert vowel articulation cannot be confirmed from this analysis, our results are consistent with existing findings on phonological processing during language production. Several studies using fMRI reported significant activation of BA46 (left MFG) during phonological processing compared to semantic processing (Price and Friston, 1997; Heim et al., 2003). Considering that our study used auditory stimuli and covert articulation of single vowels, the observed higher contribution in left MFG is reasonable.

The right IFG has been shown to have significant activation during vowel speech production compared to listening to vowel production as well as to rest (Behroozmand et al., 2015). Our experimental tasks require similar brain activity, calling for vowel speech production and listening processes. Moreover, IFG includes Broca's area, which is a well-known area of language processing, and studies have shown that these adjacent regions are involved in spoken language processing (Hillert and Buracas, 2009), selective processing of text and speech (Vorobyev et al., 2004), and voice-based inference (Tesink et al., 2009). Considering these findings, the higher contribution of right IFG in our study may be due to our requiring selective imagery of vowel sounds rather than text of vowels.

The higher contribution of left PM (BA6) seems reasonable because PM is included in speech motor areas. Speech motor areas are thought to mediate the effect of visual speech cues on auditory processing and to be associated with phonetic perception (Skipper et al., 2007; Alho et al., 2012; Chu et al., 2013). Left ITG (BA20) is often reported in studies using comprehension tasks (Papathanassiou et al., 2000; Halai et al., 2015). Considering the simplicity of our experimental tasks, the higher contribution of left ITG might be associated with selective processing of text and speech (Vorobyev et al., 2004) because we asked participants to imagine the sound of the vowel speech they heard rather than text. Our functional connectivity analysis (Figure 9) showed negative correlations between some FSHV-vertices in BA6 and other vertices in temporal areas, especially in participants who showed high classification accuracies. These findings also may support presence of selective processing of text and speech in BA20 using information from BA6 before speech production processes are initiated in the frontal areas.

### Use of SLR for classification analysis

This study employed SLR for classification analysis mainly for two reasons. One reason was simply that it offered higher classification accuracies than conventional methods, such as support vector machines (SVMs). An SVM may give better results if an effective feature selection method is applied before SVM analysis. However, we sought to minimize the number of processes to ensure robustness in BCI application. Therefore, we used SLR since it can train high dimensional classifiers without prior feature selection to reduce dimensionality, which was the second reason for its use in this study.

Owing to the ability of SLR to train high dimensional weight values without dimensional reduction, we were able to compare contribution of each brain region to classification. Analysis of mean weight values (Figure 7) showed that the areas highly contributive to classification were consistent with existing findings on the neural network for language processing. Furthermore, several studies, including a neurophysiological non-human study, have shown the validity of SLR's feature selection method, called automatic relevance determination (Mackay, 1992), for neuroscientific applications (Miyawaki et al., 2008; Tin et al., 2008; Yamashita et al., 2008). Our results offer additional evidence in support of SLR.

### Challenges and future prospects toward a BCI speller

Two steps remain for establishing an active BCI speller. First, classification accuracy must be increased for practical use. Second, the number of syllables to be classified must be increased. This study used vowels /a/ and /i/ to examine the possibility to classify Japanese words “Yes” (hái) and “No” (i.e.,). Mean accuracies of 2-class classification in this study were 82.5% (for “Group” prior) and 87.7% (for “Bestcond”), which are above the 70% accuracy deemed sufficient for real-time application (Pfurtscheller et al., 2005). Moreover, a BCI classifying “Yes” and “No” could be applicable not only as a speller but also in other applications such as cursor or robot control. For an ideal Japanese BCI speller, however, it is desirable to classify at least a 50-character syllabary including vowels and consonants. Since even existing reactive BCI spellers using stimulus-evoked potentials have difficulty in classifying 50 commands, a realistic solution would be to devise an application paradigm that includes 50 syllables but uses a smaller number of classifications (Fazel-Rezai and Abhari, 2008; Treder and Blankertz, 2010). To increase classification accuracy, we may need more parameter tuning at the individual level, because Bestcond showed that highest accuracies and contributive areas differed between participants. This study employed standard VBMEG toolbox procedures for cortical current estimation, which used SPM analysis results as priors. We varied the area and activity priors by defining thresholds in SPM statistical analysis to find the best priors for each participant. In a separate analysis, we also found a significant correlation between the number of cortical vertices and accuracy (*R* = 0.73, *p* = 0.016). This shows that it might be worthwhile to increase the number of cortical vertices not only by tuning thresholds in SPM but also by introducing anatomical information to cover full areas associated with language and speech.

## Conclusion

This study provides the first demonstration of covert vowel articulation classification using EEG cortical currents. The classification accuracy using EEG cortical currents was significantly higher than that using EEG and comparable to existing findings using semi-invasive ECoG signals. SLR weight analysis further revealed that highly contributive brain regions were consistent with the results of existing findings on language processing. With fMRI data acquisition required only once in advance to calculate an inverse filter, EEG cortical currents are a potentially effective modality for active BCI spellers.

## Author contributions

NY designed the study, performed experiments, analyses, and literature review, and drafted the manuscript. AN developed experimental programs and performed experiments and analysis. AB developed experimental programs. DS, HK performed the interventions and analyzed the results. TH, YK supervised the research and revised the manuscript. All the authors read and approved the final manuscript.

### Conflict of interest statement

The authors declare that the research was conducted in the absence of any commercial or financial relationships that could be construed as a potential conflict of interest.
